# Foot-and-mouth disease in Armenia (1958–2003): Historical epidemiology, serotype dynamics, and evolving vaccination strategies

**DOI:** 10.14202/vetworld.2025.2650-2662

**Published:** 2025-09-11

**Authors:** Henrik Voskanyan, Liana Simonyan, Nelli Shahazizyan, Mariam Mirzoyan, Jon Simonyan, Tigran Markosyan

**Affiliations:** 1 Scientific Centre for Risk Assessment and Analysis in Food Safety Area, CJSC, Yerevan 0072, Republic of Armenia; 2 Department of Livestock Breeding, Ministry of Economy, Yerevan 0010, Republic of Armenia

**Keywords:** Armenia, foot-and-mouth disease, outbreak history, progressive control pathway-foot-and-mouth disease, serotype, vaccination strategy

## Abstract

**Background and Aim:**

Foot-and-mouth disease (FMD) is a highly contagious transboundary animal disease affecting cloven-hoofed livestock, with significant economic and trade implications. Armenia lies within the West Eurasia and Middle East epidemiological pool, where serotypes O, A, Asia-1, and occasionally SAT-2 circulate. Despite decades of control efforts, the historical epidemiology of FMD in Armenia has not been comprehensively documented. This study aimed to conduct a 65-year retrospective analysis of FMD in Armenia to characterize serotype distribution, outbreak patterns, vaccination strategies, and diagnostic advancements, and to identify priorities for progression in the progressive control pathway (PCP-FMD).

**Materials and Methods:**

Data from 1958 to 2023 were compiled from the Union of Soviet Socialist Republics (USSR) agricultural archives, national veterinary records, World Organization for Animal Health/World Reference Laboratory for FMD reports, and peer-reviewed literature. Serotype identification, outbreak frequency, and species involvement were analyzed using descriptive statistics, heatmaps, and geographic information system (GIS) mapping. Diagnostic evolution from complement fixation testing to enzyme-linked immunosorbent assay, reverse transcription-polymerase chain reaction, and viral protein 1 (VP1) sequencing was documented. Vaccination protocols were traced from early monovalent campaigns to current polyvalent strategies.

**Results:**

Between 1958 and 2023, Armenia recorded over 1 million FMD cases, with peaks in 1966 (591,820 cases) and 1973 (471,263 cases). Serotypes O, A, Asia-1, and SAT-1 were detected, with serotype O predominating. Outbreaks declined significantly after the 1980s, coinciding with mass vaccination, improved diagnostics, and targeted biosecurity measures. Notable milestones included integration of the A/Armenia/98 strain into vaccines (1999) and adoption of polyvalent vaccines containing the A/ASIA/G-VII lineage (2016). No outbreaks have been reported since 2016.

**Conclusion:**

Armenia’s sustained control of FMD reflects adaptive vaccination strategies, early serotype detection, and regional cooperation. Progression from PCP-FMD Stage 2 to Stage 3 will require enhanced vaccination coverage, expanded surveillance, and strengthened veterinary infrastructure. Historical lessons from Armenia’s control strategies may inform FMD management in similar transboundary risk zones.

## INTRODUCTION

Foot-and-mouth disease (FMD) is a highly contagious viral infection that affects cloven-hoofed animals, including cattle, buffalo, pigs, sheep, goats, and approximately 70 species of wildlife. It is present in nearly every region where livestock are raised, with more than 100 countries currently or historically affected [1]. In nations typically free from FMD, outbreaks can cause severe economic losses, while in endemic regions the disease reduces animal productivity and imposes trade restrictions on animal products [2].

The causative agent, FMD virus (FMDV), is a single-stranded, positive-sense RNA virus of the genus *Aphthovirus* in the family *Picornaviridae*. FMDV is classified into seven serotypes, A, O, C, Asia-1, SAT-1, SAT-2, and SAT-3, each containing multiple strains or lineages [3]. Infected animals are the primary source of the virus, shedding large quantities during the acute phase through aerosolized exhalations, as well as saliva, feces, and urine [4].

Serotype C has not been detected since 2004, following its last recorded outbreaks in Brazil and Kenya. The remaining six serotypes still circulate globally [5], distributed across seven regional pools that reflect distinct geographical and epidemiological patterns [6]. This classification, established by the World Organization for Animal Health (WOAH) and the Food and Agriculture Organization (FAO), supports targeted vaccine development and coordinated control strategies. The pools are as follows: Pool 1 - Southeast Asia, East Asia, and Central Asia; Pool 2 - South Asia; Pool 3 - West Eurasia and the Middle East; Pool 4 - Eastern Africa; Pool 5 - West and Central Africa; Pool 6 - Southern Africa; and Pool 7 - South America [7].

Armenia belongs to Pool 3, where the predominant circulating serotypes are O, A, Asia-1, and occasionally SAT-2, with distribution varying by country and over time [8–10]. The nation’s livestock sector, comprising cattle, sheep, and pigs, plays a critical role in food security and local economies. However, Armenia remains highly vulnerable to FMD introduction and spread due to its proximity to endemic countries and frequent cross-border livestock movement.

The progressive control pathway for FMD (PCP-FMD), developed by WOAH and FAO, provides a structured framework to help countries evaluate and strengthen their FMD control programs. It outlines five stages, from understanding disease epidemiology to achieving and maintaining FMD-free status [1]. Armenia is currently in Stage 2, which emphasizes risk-based control measures to limit the disease’s impact in targeted livestock sectors. Key priorities include identifying high-risk areas and practices, expanding vaccination coverage, and strengthening surveillance and reporting systems [11].

To support these efforts, the FAO and the European Commission for the Control of FMD have developed the West Eurasia Regional Roadmap for Progressive Control of FMD. This long-term strategy aligns with the PCP-FMD framework and promotes regional collaboration, enhanced technical capacity, and the implementation of science-based control measures.

Although FMD remains one of the most significant transboundary animal diseases globally, long-term, country-specific epidemiological records are often fragmented, particularly in regions undergoing major political, economic, and institutional transitions. In the case of Armenia, historical data from the Soviet era, the post-independence period, and the modern surveillance framework have never been comprehensively integrated into a single longitudinal analysis. Existing regional studies often combine Armenia’s data with those of neighboring countries, obscuring country-level trends in serotype distribution, outbreak dynamics, and the impact of evolving vaccination strategies. Furthermore, most reports focus on short-term outbreak investigations or vaccine evaluations, with limited attention to multi-decade changes in epidemiology, diagnostic capabilities, and control measures. The absence of such a consolidated historical perspective hinders the ability to fully assess Armenia’s progress along the PCP-FMD and limits the evidence base for designing targeted, risk-based interventions in the context of current transboundary threats.

This study aimed to conduct a comprehensive retrospective and historical analysis of FMD in Armenia over 65 years (1958–2023), integrating archival, national, regional, and international data sources. Specifically, the study sought to: (i) document the temporal and spatial patterns of FMD outbreaks across different political eras; (ii) characterize the distribution of FMDV serotypes and their relationship to regional circulation patterns; (iii) evaluate the evolution of diagnostic methods and vaccination strategies in response to changing epidemiological conditions; and (iv) identify key milestones, persistent vulnerabilities, and priority actions required for Armenia’s progression from PCP-FMD Stage 2 to Stage 3. By bridging historical epidemiology with contemporary control frameworks, this work aims to inform both national and regional strategies for sustainable FMD prevention and eventual eradication.

## MATERIALS AND METHODS

### Ethical approval

This retrospective study involved no live animal handling. All data were obtained from publicly available databases, historical records, and authorized institutional archives. While formal ethical approval was not required, the Ministry of Agriculture and the Food Safety Inspection Body of Armenia granted permission for data access.

### Study period and location

The study was conducted from the early 1990s (initial data extraction), with the main data analysis and manuscript preparation carried out from 2022 onward. Armenia is a landlocked country in the South Caucasus region of Eurasia, positioned at the intersection of Eastern Europe and Western Asia. It shares borders with Turkey to the west, Georgia to the north, Azerbaijan to the east, and Iran to the south. Geographically, Armenia spans approximately 38°–42° N latitude and 43°–47° E longitude. The terrain is predominantly mountainous, characterized by mountain ranges, high plateaus, and deep valleys, with an average elevation of around 1,800 m (5,900 feet) above sea level. These diverse landscapes influence agricultural and livestock practices across the country.

### Livestock demographics and susceptible populations

As of January 1, 2024, livestock populations in Armenia susceptible to FMD were as follows:

- Cattle: 491,594 head
- Small ruminants (SR) (sheep and goats): 690,565 head
- Pigs: 186,951 head.

These figures are derived from national veterinary statistical reports and reflect the populations under FMD risk surveillance.

### Data sources and timeframes

#### Archival period (1958–1991)

- USSR Ministry of Agriculture reports: Historical outbreak data were obtained from Soviet archives in Moscow, documenting suspected and confirmed FMD cases. Laboratory confirmation was limited during large outbreaks due to high volume of cases.

#### Post-independence records (1991–2016)

- National veterinary records: Annual epidemiological reports and official veterinary documents from the Ministry of Agriculture and the Food Safety Inspection Body of Armenia provided detailed outbreak data following independence in 1991.

#### Scientific and laboratory data (1958–2023)

- Data from the Transcaucasian Foodborne Disease Institute and the Scientific Center for Risk Assessment and Analysis in Food Safety were used to track diagnostic and epidemiological changes over time.

#### International reports (1991–2023)

- WOAH (World Animal Health Information System): FMD data from 1996 to 2023
- World reference laboratory for FMD (WRLFMD): Data from 1957 to 2023
- All-Russian Research Institute for Animal Health - ARRIAH (Russia): Genotyping and molecular epidemiology records.

### Literature review

A comprehensive search was performed in Web of Science, PubMed, Scopus, Google Scholar, Mendeley, and the Russian Scientific Citation Database to identify peer-reviewed studies and historical reports relevant to FMD in Armenia. A comprehensive search was performed using broad and iterative keyword combinations related to “foot-and-mouth disease” and “Armenia,” including terms such as “FMD,” “serotype,” “outbreak,” and “vaccination.”

### Diagnostic methods and standards for confirmation

#### Soviet era (1958–1991)

FMD diagnosis relied on a combination of clinical signs (e.g., vesicular lesions, salivation, lameness), epidemiological data, and laboratory testing where feasible. Suspected cases included animals from affected herds without laboratory confirmation. The complement fixation test (CFT) was the primary laboratory tool for diagnosing and serotyping FMDV [12].

#### Post-independence developments (1998–2015)

Advancements in laboratory diagnostics included:

- Enzyme-linked immunosorbent assay (ELISA) for antigen detection, using bovine epithelium samples cultured in bovine thyroid and porcine kidney epithelial (IB-RS-2) cell lines [13, 14].
- Reverse transcription-polymerase chain reaction for molecular detection.
- *VP1* gene sequencing for molecular epidemiology of selected samples (2000–2015) [15].

#### Current standards

Armenia now follows WOAH-recommended protocols for FMD case definitions and laboratory confirmation, as outlined in the Terrestrial Animal Health Code, Chapter 8.8, Article 8.8.1, point 3 [16].

### Statistical and GIS analyses

- Descriptive statistics summarized the temporal and regional distribution of FMD cases.
- Cross-tabulations organized data by species, serotype, and year [17].
- Heatmaps (Microsoft Excel) illustrated outbreak intensity, with thresholds set at <100, 100–1,000, >1,000, >10,000, and >100,000 cases [18].
- QGIS v3.40 LTR (https://www.qgis.org) mapped outbreak density and serotype distribution. Transmission corridors were visualized by overlaying Marz-level administrative boundaries with road and river networks from OpenStreetMap (OpenStreetMap contributors) and Natural Earth databases via QuickOSM (QGIS Development Team, https://www.qgis.org).

### Data inclusion and reliability

Records were included if they reported suspected or confirmed FMD cases in Armenia and provided at least one key detail (year, location, or affected species).

- Exclusion criteria: Records lacking temporal or spatial data, unverifiable through official archives.
- Historical adjustments: Pre-1991 records lacking laboratory confirmation were categorized as suspected cases and analyzed separately.
- Quality control: Data were retrospectively aligned with WOAH standards where possible, and inconsistent or incomplete entries were flagged. While early records varied in reliability, multi-source verification enhanced the robustness of the dataset.

## RESULTS

### Pre-serotyping era (1958–1965)

#### First recorded cases

FMD was first officially reported in Armenia in 1958. At this time, FMD serotyping was not routinely performed within the country. Diagnosis and case definitions were primarily based on clinical signs, such as vesicles on the tongue, gums, lips, and other parts of the oral cavity, salivation, and lameness, and on epidemiological data, including prevalence, incidence, geographical distribution, and vaccination coverage. Laboratory confirmation was rare.

Between 1958 and 1960, the USSR Ministry of Agriculture introduced regulations to establish a specialized FMD institute, which included an experimental laboratory dedicated to vaccine development.

### Available data and serotype detection

**Figure 1:**
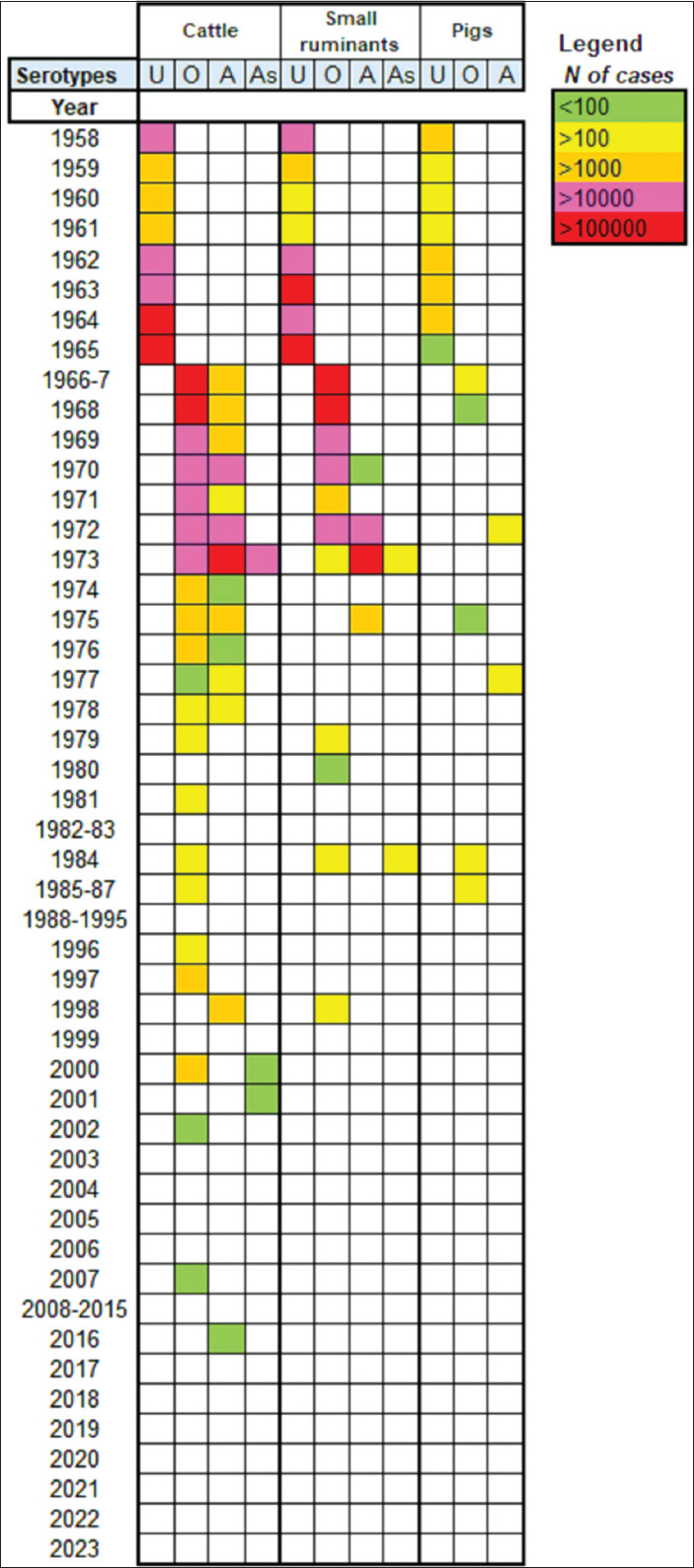
Prevalence of foot-and-mouth disease virus Serotypes by Livestock Species and Year (1958–2023). Serotypes U (Unknown) and As (Asia-1).

From 1958 to 1965, annual reports from the USSR Ministry of Agriculture primarily recorded the number and species of livestock affected (Figure 1). In 1962, the All-Union Scientific Research Institute (AUSRI) for FMD was established in Vladimir, Russia, and became fully operational. Only a small number of infected animal samples from Armenia were sent to AUSRI for serotyping.

**Figure 2:**
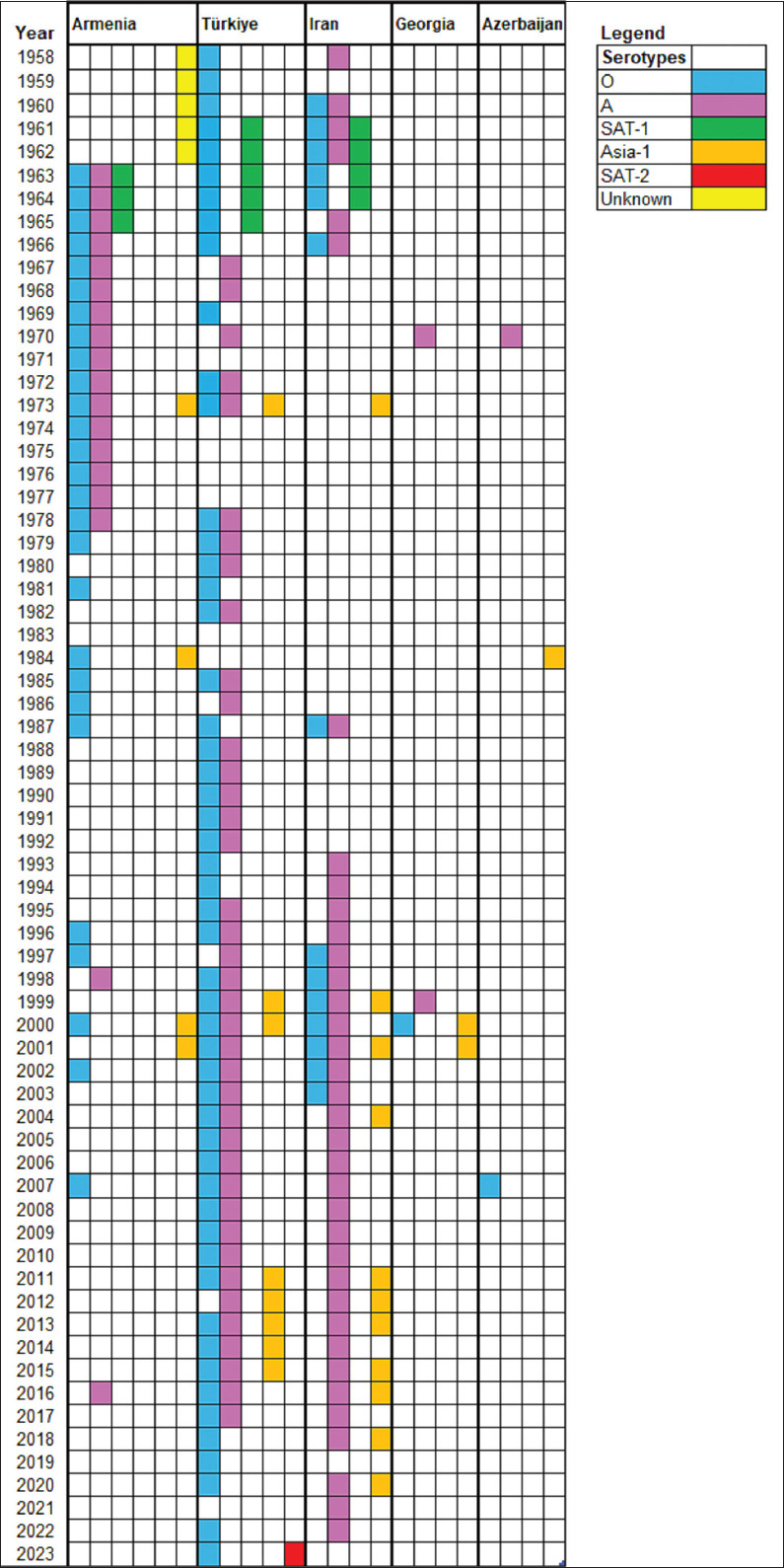
Chronological patterns of foot-and-mouth disease virus serotypes in Armenia and Adjacent Countries (1958–2023).

Between 1963 and 1965, serotypes A, O, and SAT-1 were identified in Armenia. During 1962–1965, approximately 93% of cases occurred in cattle, 85% in SR, and 65% in pigs. WRLFMD data [19] also confirmed the detection of these serotypes in Armenia’s neighboring countries during the same period (Figure 2).

### Vaccination strategies

From 1958, FMD vaccines in the USSR targeted monovalent formulations against serotypes A and O, with occasional adjustments for other prevalent strains. In Armenia, these vaccines were routinely administered to cattle and SR, while pigs were vaccinated only in outbreak areas. Between 1963 and 1965, SAT-1 vaccination was also implemented.

In 1965, the USSR Ministry of Agriculture issued the “Instruction on Measures for the Prevention and Eradication of FMD in Farm Animals” [19], outlining quarantine protocols, epizootic zone management, animal transport precautions, and post-quarantine restrictions.

### Soviet diagnostic and control expansion era (1966–1991)

#### Improved diagnostics

From 1966, Armenia began conducting in-country FMD serotyping using the CFT, a method that remained in use until 1991. This marked a major advancement in outbreak reporting and laboratory confirmation.

#### Epidemiological trends (1966–1981)

**Figure 3:**
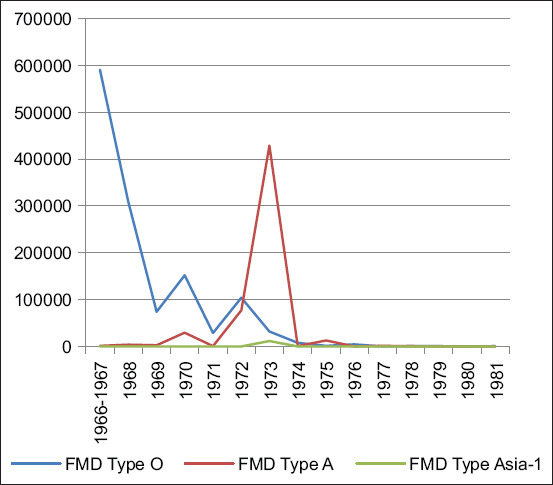
Total number of foot-and-mouth disease Cases by Serotype (1966–1981).

**Figure 4:**
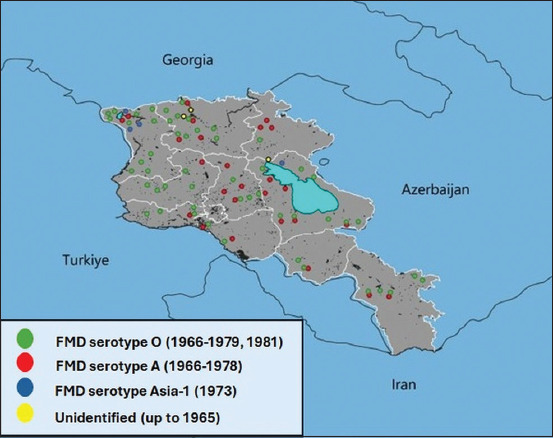
Outbreaks of foot-and-mouth disease in the Republic of Armenia (1965–1981).

FMD cases were recorded annually, with serotypes O and A predominating between 1966 and 1973. The highest recorded case numbers were 591,820 in 1966 and 471,263 in 1973. The Asia-1 serotype was first reported in Armenia in 1973. After 1973, only serotypes O and A were detected, and case numbers dropped substantially (Figure 1). Geographical distribution data for some serotypes became available from 1973 onward (Figures 3 and 4).

#### Epidemiological trends (1982–1991)

**Figure 5:**
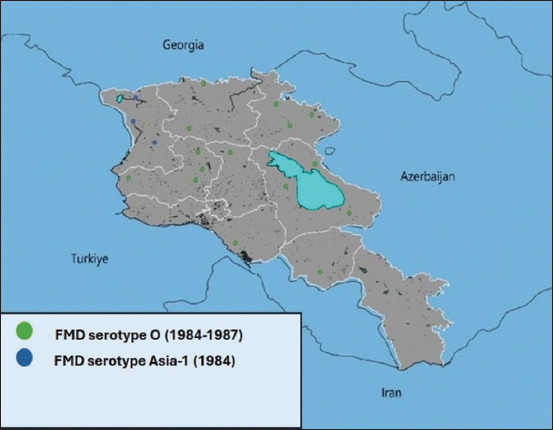
Outbreaks of foot-and-mouth disease in the Republic of Armenia (1984–1987).

Between 1982 and 1991, FMD incidence decreased significantly. In 1984, cases involved serotype O (cattle: 985; SR: 530; pigs: 334) and Asia-1 (SR: 210). From 1985 to 1987, only serotype O was detected (cattle: 138; pigs: 130) (Figure 5). WRLFMD records show similar trends in neighboring Turkey and Iran, including a shared Asia-1 outbreak in 1973 (Figure 2).

#### Vaccination strategies

In 1966, the USSR launched a coordinated FMD eradication program [20], involving 16 research institutions, including the Armenian Research Institute of Veterinary and Livestock Breeding. By 1970, two regional branches were operational: the Transcaucasian Branch in Yerevan and the Middle Eastern Branch in Dushanbe.

Routine vaccination in Armenia used monovalent vaccines for serotypes A and O. Between 1973 and 1987, Asia-1 vaccination was added. In 1978, a polyvalent vaccine (A, O, and C) was introduced (Table 1). Legislative updates in 1985 [21] formalized modernized protocols for sample collection, serotyping, vaccine production, and application.

**Table 1:** Chronological summary of the epidemiological events and vaccination strategies of FMD in Armenia (1958–2023).

Year	Event	Vaccination strategy
USSR era (up to 1991)		
1958	First FMD reports in Armenia	Using of monovalent vaccines (A, O) begin
1959–1962	FMD outbreaks (unidentified serotypes)	Monovalent vaccination (A, O)
1963–1965	All-Union Scientific	SAT-1 vaccine introduced in parallel with monovalent (A, O)
1966	Serotyping started. A, O, SAT-1 serotypes identified	
1967–1972	CFT serotyping in Armenia started. Pick of cases (591,820) A, O	Monovalent vaccination (A, O)
1973	FMD annual outbreaks (A, O)	Monovalent vaccination (A, O)
1974–1977	First cases of Asia-1 identified with A, O. Pick of cases (471,263)	Monovalent vaccination (A, O)
1978	FMD annual outbreaks (A, O)	Monovalent vaccination (A, O)
1979–1981	FMD outbreaks (A, O)	Polyvalent vaccine (A, O, C) with monovalent (Asia-1)
1982–1983 1984	Limited FMD outbreaks (O) First time no FMD cases/outbreaks registered	Polyvalent vaccine (A, O, C) with monovalent (Asia-1) Polyvalent vaccine (A, O, C) with monovalent (Asia-1)
1985–1987	Limited FMD outbreaks (Asia-1, O)	Polyvalent vaccine (A, O, C) with monovalent (Asia-1)
1988–1991	Limited FMD outbreaks (O)	Polyvalent vaccine (A, O, C) with monovalent (Asia-1)
	No FMD cases/outbreaks registered	Polyvalent vaccine (A, O)
Post - independence era (starting from 1991)		
1991–1995	No FMD cases/outbreaks officially registered	Polyvalent vaccine (A, O)
1996–1997	First post-independence FMD outbreaks (O)	Polyvalent vaccine (A, O)
1998	FMD outbreaks (A/Armenia/98 strain)	Polyvalent vaccine (A, O) with A/Armenia/98 strain included
1999	No FMD cases/outbreaks registered	Polyvalent vaccine (A/Armenia/98, O)
2000–2001	FMD outbreaks (O, Asia-1)	Polyvalent vaccine (A/Armenia/98, O, Asia-1)
2002	Limited FMD outbreaks (O)	Polyvalent vaccine (A/Armenia/98, O, Asia-1)
2003–2006	No FMD cases/outbreaks registered	Polyvalent vaccine (A, O, Asia-1)
2007	Limited FMD outbreaks (O)	Polyvalent vaccine (A, O, Asia-1)
2008–2015	No FMD cases/outbreaks registered	Polyvalent vaccine (A, O, Asia-1)
2016	FMD outbreak (A – G-VII lineage)	Polyvalent vaccine (A + AG-VII, O, Asia-1)
2017–2022	No FMD cases/outbreaks registered	Polyvalent vaccine (A + AG-VII, O, Asia-1)
2023	No FMD cases/outbreaks registered	Polyvalent vaccine (A + AG-VII, O, Asia-1, SAT-2)

FMD = Foot-and-mouth disease, CFT = Complement fixation test, USSR = Union of Soviet Socialist Republics

### Post-Soviet/independent Armenian era (1991–2024)

#### Early post-independence (1991–1997)

Following Armenia’s independence in 1991, the first available outbreak data are from 1992 and 1993, recording five and seven outbreaks, respectively, though without serotype information [22]. WRLFMD data indicate that serotypes A and O were present in the region at the time.

In 1996, serotype O was confirmed in cattle (198 cases), and in 1997, cases increased (cattle: 1,026; SR: 174).

#### Emergence of A/Armenia/98 strain (1998–1999)

In 1998, FMD serotype A caused 7,798 cattle cases. Laboratory analysis at ARRIAH, Russia, identified the A1707 Armenia/98 strain, closely related to A/Iran-96 and A/Türkiye-97. ARRIAH used this strain to develop a new vaccine [23], which Armenia adopted from 1999 to 2007 [24].

#### Mixed serotype outbreaks (2000–2002)

Between 2000 and 2002, outbreaks were associated with serotypes O and Asia-1. In 2000, 1,087 cattle were affected by serotype O. Asia-1 was detected in cattle in 2000–2001 (69 cases). In 2002, serotype O reappeared with 36 confirmed cases in cattle. Sequencing revealed close genetic relationships between the Armenia Asia-1 strain and strains from Türkiye and Georgia [25].

#### Sporadic outbreaks and the 2016 emergency

A small outbreak of serotype O occurred in 2007 (13 cattle cases). In 2016, a case of serotype A/ASIA/G-VII was confirmed through CFT, ELISA, and sequencing. This strain closely matched viruses from Iran and Türkiye [25]. Throughout this period, Armenia’s neighbors continued to report serotypes A, O, and Asia-1 [26, 27].

#### Vaccination strategies (1999–2024)

**Figure 6:**
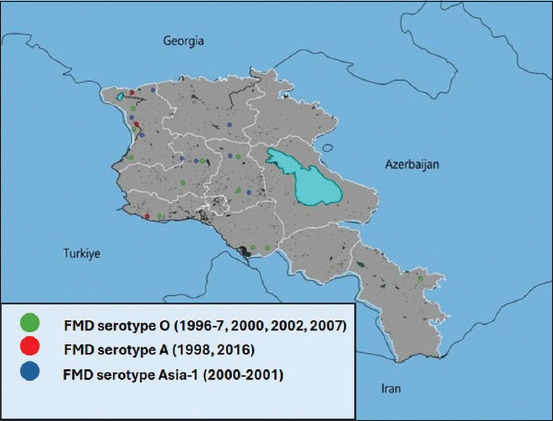
Outbreaks of foot-and-mouth disease in the Republic of Armenia (1996–2016).

In 1999, the A/Armenia/98 strain was integrated into Armenia’s routine vaccine. In 2016, the A/ASIA/G-VII lineage was added. Since then, Armenia has consistently used polyvalent vaccines, with the serotype distribution from 1996 to 2016 as shown in Figure 6 [28].

## DISCUSSION

For decades, FMD has been one of the most significant transboundary animal diseases affecting Armenia and neighboring countries. It has appeared both as widespread epidemics and as sporadic outbreaks.

### Early outbreaks and pre-vaccination strategies (1958–1965)

The first officially confirmed cases of FMD in Armenia, then part of the Soviet Union, were recorded in 1958. FMD serotyping was performed for samples from Armenia, and by 1965, serotypes A, O, and SAT-1 had been detected. WRLFMD reports confirmed the same serotypes in neighboring Iran and Türkiye between 1961 and 1965 [29].

Vaccination of cattle and SR, with limited vaccination for pigs, began in 1958. Between 1963 and 1965, bivalent A and O vaccines were used alongside a monovalent SAT-1 vaccine to control outbreaks. At that time, the USSR’s initial strategy did not involve systematic vaccination; instead, infected premises were depopulated by slaughtering all susceptible animals [30].

Disease control was further impeded by the lack of a coordinated national surveillance framework, standardized diagnostic protocols, and prompt outbreak reporting. The prevalence of collective farming, with large herds grazing openly, facilitated the rapid spread once an outbreak occurred.

### USSR eradication program and expanded vaccination (1966–1979)

In 1966, the USSR launched an FMD eradication program [20, 31] in collaboration with multiple research institutions. Armenia used vaccines derived from the A and O strains, which were considered the most immunogenic against circulating viruses in the region. This period saw a decline in outbreak frequency, although exceptions occurred.

Neighboring Iran and Türkiye also experienced outbreaks of serotypes A and O. The Asia-1 serotype was first detected regionally in 1973 but did not cause further outbreaks in Armenia due to targeted vaccination with the Asia-1 strain which was introduced that year.

### Improved control and regional collaboration (1980–1990)

The 1980s brought substantial improvement in Armenia’s FMD status. Outbreak numbers dropped significantly due to large-scale vaccination campaigns and a scientifically based control strategy [20].

Regional FMD conditions also improved. By 1987, only serotypes A and O were reported by World Organization for Animal Health in Iran [16]. Both Türkiye and Iran implemented mass vaccination strategies similar to those in Armenia. Iran introduced polyvalent vaccination and movement restrictions after incursions of Asia-1 and SAT-1 [32, 33], while Türkiye established buffer vaccination zones in Thrace and expanded to national biannual vaccination campaigns with bivalent and trivalent vaccines [34].

Armenia benefited from centralized USSR vaccine production, standardized protocols, and coordinated outbreak reporting–advantages that contributed to its relatively stable FMD situation.

#### Post-independence period and first recurrences (1991–1998)

After gaining independence in 1991, Armenia’s veterinary services and livestock systems changed, but biannual vaccination against serotypes A and O continued. The stable situation achieved in the late 1980s persisted initially, but the first post-independence FMD cases were reported in 1996–1997, involving serotype O in cattle and SR.

In 1998, outbreaks of serotype A occurred in cattle, including vaccinated herds. Laboratory analysis of isolates (A/Armenia/98) revealed genetic differences from A/Türkiye/98, A/Türkiye/97, and A/Iran/96 strains [23].

### Local vaccine development and new serotype incursions (1999–2007)

Following the 1998 outbreak, Armenia initiated local vaccine production, incorporating the A/Armenia/98 strain into a bivalent A and O vaccine that was used from 1999 onward.

From 1999, the Asia-1 serotype, which had been absent in the region since 1973, was reintroduced in Türkiye and Iran [35]. Armenia recorded Asia-1 outbreaks in 2000–2001 [36, 37]. The As1/Armenia/2000 strain shared 99.18% similarity with Turkish isolates [25].

In response, Armenia adopted a trivalent vaccine (A, O, Asia-1) from 2000 until 2007, when local vaccine production ceased. From then until 2007, FMD incidence remained low, with only sporadic outbreaks in 2002 and 2007 (both serotype O).

### Regional threats and emergency response (2015–2016)

In 2015, the A/ASIA/G-VII lineage spread rapidly across the region, causing multiple outbreaks [9]. Armenia confirmed its first case in 2016. The virus closely matched the A/IRN/12/2015 strain [25].

This prompted the incorporation of the A/ASIA/G-VII lineage into the polyvalent vaccine produced at ARRIAH, containing A/ASIA/Iran-05, O/ME-SA/PanAsia-2, and Asia-1/ASIA/Sindh08 strains. This vaccine has been used since 2016, maintaining Armenia’s FMD-free status [38].

### Advancements in diagnostics and vaccine strategies

During the Soviet period (1958–1991), FMD diagnosis relied mainly on clinical observation and limited virological testing, with confirmation performed in centralized USSR laboratories.

After gaining independence, Armenia adopted modern diagnostic techniques, including ELISA and molecular methods, particularly after 2000, with support from international reference laboratories. Vaccine strategies progressed from monovalent/bivalent vaccines (O, A, SAT-1) to the inclusion of Asia-1 in the 1970s, the local production of a bivalent vaccine in 1999, trivalent vaccines from 2000 to 2007, and the development of a polyvalent formulation since 2016.

Serotyping improvements enhanced epidemiological resolution, enabling better outbreak tracking, vaccine matching, and regional coordination.

### Current PCP-FMD status and future directions

Armenia is currently at PCP-FMD Stage 2 [11], having established a strong understanding of FMD risks and initiated targeted control measures. To progress to Stage 3, Armenia must:

- Demonstrate measurable improvements in vaccination coverage and effectiveness
- Strengthen veterinary services with adequate resources and rapid-response capacity
- Expand systematic surveillance and timely reporting
- Increase farmer participation in disease prevention.

A comprehensive monitoring and evaluation system is also essential to assess the effectiveness of control measures and to modify strategies as needed. Achieving these objectives would enable Armenia to progressively eliminate FMD within designated zones, moving toward regional freedom from the disease.

## CONCLUSION

This study provides the first comprehensive, long-term analysis of FMD in Armenia, spanning 65 years (1958–2023) and integrating national, regional, and international data sources. The historical review revealed that four FMDV serotypes, O, A, Asia-1, and SAT-1, have circulated in Armenia, with serotype O being the predominant one. The highest outbreak peaks occurred in 1966 (591,820 cases) and 1973 (471,263 cases), followed by a marked decline in incidence due to systematic mass vaccination, targeted strain inclusion (e.g., A/Armenia/98 and A/ASIA/G-VII), and improved diagnostic capabilities. No outbreaks have been recorded since 2016, highlighting the effectiveness of the polyvalent vaccine currently in use.

The findings highlight the importance of adaptive, strain-specific vaccination strategies, ongoing serotype monitoring, and robust regional cooperation in achieving long-term control of transboundary animal diseases. The historical data offer a valuable evidence base for refining Armenia’s PCP-FMD strategy, guiding vaccine selection, and improving preparedness for potential reintroductions from endemic neighboring regions.

One of the key strengths of this study is its use of one of the longest continuous national FMD datasets in the region, validated with WRLFMD and WOAH records. It integrates historical, epidemiological, molecular, and vaccination data into a unified temporal framework and clearly demonstrates how vaccine strain updates aligned with regional epidemiology contributed to sustained control.

However, certain limitations exist. Early Soviet-era records lacked laboratory confirmation and detailed geographic resolution, limiting retrospective serotype mapping before 1966. Variability in reporting quality between political eras may have influenced temporal outbreak comparisons, and the limited availability of molecular sequence data for some historical isolates restricts detailed phylogenetic analysis.

Future progress from PCP-FMD Stage 2 to Stage 3 will require expanding vaccination coverage and ensuring cold-chain reliability in remote areas, enhancing post-vaccination monitoring to assess immune protection levels, strengthening cross-border surveillance and coordinated response mechanisms with Türkiye, Iran, and Georgia, expanding molecular epidemiology studies to detect emerging antigenic variants early, and leveraging GIS-based risk mapping to target resources to high-risk zones.

Armenia’s experience demonstrates that sustained commitment to science-based vaccination, early detection, and regional collaboration can transition a country from recurrent epidemics to a stable, near-elimination status. By addressing the identified gaps in surveillance, farmer engagement, and veterinary infrastructure, Armenia is well-positioned to progress toward FMD-free status, contributing to the broader regional and global objective of FMD eradication.

## DATA AVAILABILITY

Archived data are available at the Scientific Center in paper format in Russian and Armenian, encompassing reports, maps, and various types of scientific publications. The corresponding author can provide the archived data from the Scientific Center on a reasonable request.

## AUTHORS’ CONTRIBUTIONS

HV: Conceptualized the study and collected historical data. TM, LS, NS, MM, and JS: Contributed to data collection and manuscript revision. TM and JS: Performed data extraction, developed maps and graphics, and conducted statistical analyses. TM: Drafted the manuscript. All authors reviewed and approved the final version of the manuscript.
